# Supplementary material for: Real-world before-and-after evaluation of AI support for lung cancer diagnosis at three US lung nodule clinics
Source: PLoS One. 2026 Oct 14;21(10):e0358655. doi: 10.1371/journal.pone.0358655 (PMC13667789; doi:10.1371/journal.pone.0358655)
Supplement: Supplementary file 1 [file pone.0358655.s001.pdf]

S1 Table. Site-level outcomes before and after VNC implementation

Site-level results for new-patient volumes, procedures, confirmed lung cancer diagnoses, early-stage lung cancer diagnoses, and time from baseline CT to confirmed cancer diagnosis before and after VNC implementation.

Outcome	Site A		Site B		Site C		Total	
	Before VNC	After VNC	Before VNC	After VNC	Before VNC	After VNC	Before VNC	After VNC
Paired months	5.0	5.0	4.5	4.5	3.0	3.0	12.5	12.5
New patients	32	74	56	76	50	82	138	232
Patients undergoing ≥1 invasive diagnostic procedure	10	24	25	35	26	41	61	100
Confirmed lung cancers	3	10	19	17	15	21	37	48
Early-stage lung cancers	1	8	10	11	7	11	18	30
Cancer cases included in time-to-diagnosis analysis	3	10	19	14	15	21	37	45
Time from baseline CT to confirmed cancer diagnosis, median [IQR], days	58 [45.5–151.0]	52 [20.3–69.5]	46 [33.5–132.0]	60 [44.8–104.3]	65 [42.5–202.0]	52 [26.0–105.0]	58 [33–158]	57 [27–85]

Notes:

Values are counts unless otherwise specified. Site A was a regional health system hospital, Site B was a community hospital, and Site C was an academic medical center.

Procedures correspond to patients undergoing at least one percutaneous or bronchoscopic biopsy, or surgical resection, within the matched follow-up window.

Early-stage cancer refers to cancers diagnosed at stages I–II. CT, computed tomography; IQR, interquartile range; VNC, Virtual Nodule Clinic.
