# Supplementary material for: Real-world before-and-after evaluation of AI support for lung cancer diagnosis at three US lung nodule clinics
Source: PLoS One. 2026 Oct 14;21(10):e0358655. doi: 10.1371/journal.pone.0358655 (PMC13667789; doi:10.1371/journal.pone.0358655)
Supplement: Supplementary file 2 [file pone.0358655.s002.pdf]

S2 Table. Site-level contextual factors and potential confounders

Summary of available site-level contextual factors before and after VNC implementation.

Contextual factor	Site A		Site B		Site C	
	Before VNC	After VNC	Before VNC	After VNC	Before VNC	After VNC
**Setting**	Regional health system hospital	Regional health system hospital	Community hospital (thoracic surgery clinic)	Community hospital (thoracic surgery clinic)	Academic medical center	Academic medical center
**Clinic leadership**	Nurse practitioner and interventional pulmonologist	Nurse practitioner and interventional pulmonologist	Nurse practitioner, thoracic surgeon, and nurse navigator	Nurse practitioner, thoracic surgeon, and nurse navigator	Advanced practice provider and interventional pulmonologist	Advanced practice provider and interventional pulmonologist
**Patient identification sources**	External referrals only	AI-enabled discovery and external referrals	External referrals and manual internal electronic-record flagging	AI-enabled discovery and external referrals	External referrals only	AI-enabled discovery and external referrals
**Referral workflow**	Standard referral to nodule clinic	AI-discovered patients referred after outreach to the primary care provider, referring provider, or patient	Standard referral to nodule clinic	AI-discovered patients referred after outreach to the primary care provider, referring provider, or patient; some low-risk patients referred to pulmonology rather than surgery	Standard referral to nodule clinic	AI-discovered patients referred after outreach to the primary care provider, referring provider, or patient
**Staffing**	Nurse practitioner and interventional pulmonology team	Nurse practitioner and interventional pulmonology team	Nurse navigator, nurse practitioner, and thoracic surgeon	Nurse navigator, nurse practitioner, and thoracic surgeon	Advanced practice provider and interventional pulmonology team	Advanced practice provider and interventional pulmonology team
**Lung cancer screening activity**	Active	Active	Active	Active	Active	Active
**Clinic capacity**	Not tracked	Not tracked	Not tracked	Not tracked	Not tracked	Not tracked
**Imaging volume**	Not tracked	Not tracked	Not tracked	Not tracked	Not tracked	Not tracked
**Diagnostic / discovery criteria**	Referred patients	AI-enabled discovery threshold of 6 mm, plus referred patients	Referred patients	AI-enabled discovery threshold of 8 mm, plus referred patients	Referred patients	AI-enabled discovery threshold of 6 mm, plus referred patients
**Other contextual changes**	None identified from available site-level inputs	None identified from available site-level inputs	None identified from available site-level inputs	Loss of a key cancer referral source during the post-implementation period	None identified from available site-level inputs	None identified from available site-level inputs

Notes:

This table summarizes available site-level contextual factors before and after implementation. It is descriptive and does not provide adjusted estimates.

AI-enabled discovery thresholds refer to the nodule-size thresholds used for automated case discovery.

EMR, electronic medical record; mm, millimeter.
